# *Toxoplasma gondii* in Ethiopia: A Systematic Review and Meta‐Analysis of Infection Prevalence and Associated Risk Factors in Humans and Animals

**DOI:** 10.1002/vms3.71293

**Published:** 2026-11-03

**Authors:** Ambachew Motbaynor Wubaye, Birhanu Feleke Shitu, Amare Mebrat Delie, Aysheshim Belaineh Haimanot, Yezbie Wagaw Ayicheh, Adugna Mengesha Mulugeta, Anley Shiferaw Enawgaw, Yihenew Getahun Ambaw, Mequanent Dessie Bitewa, Smegnew Gichew Wondie, Tadele Derbew Kassie, Zinie Abita Mengie, Simegnew Adugna Kallu, Berhanu Ayana Tafere, Tesfaye Shumet Mekonnen, Mikias Getahun Molla, Daniel Mulat Eshetu, Thomas Kidanemariam Yewodiaw

**Affiliations:** ^1^ Department of Veterinary Science, College of Agriculture and Environmental Science Debre Tabor University Debre Tabor Ethiopia; ^2^ Northern Resilience Cluster Office Jhpiego Ethiopia; ^3^ Department of Public Health, College of Medicine and Health Sciences Injibara University Injibara Ethiopia; ^4^ Department of Veterinary Medicine, College of Agricultural Sciences Woldia University Woldia Ethiopia; ^5^ College of Veterinary Medicine Haramaya University Dire Dawa Ethiopia; ^6^ Department of Midwifery, College of Medicine and Health Sciences Debre Markos University Debre Markos Ethiopia; ^7^ Medical Officer at International Medical Corps Amhara Region Emergency Operation Center, Gondar Field Office Gondar Ethiopia

**Keywords:** Ethiopia, human, prevalence, ruminants, *Toxoplasma gondii*

## Abstract

*Toxoplasma gondii* is a protozoan parasite with significant animal and public health implications. The aim of this systematic review and meta‐analysis was to estimate the pooled prevalence of toxoplasmosis in ruminants and humans in Ethiopia and to determine the major risk factors associated with the disease. Five databases were used to search for all articles published between 2012 and 2025. The pooled prevalence of toxoplasmosis was calculated using a random‐effects model. *I* ^2^ statistics were used to assess heterogeneity among the included studies. Publication bias was assessed through a meta‐analysis Egger regression asymmetry test. The overall pooled prevalence of *T. gondii* was 42.0% (95% CI: 35.0%–49.0%) in ruminants and 61.0% (95% CI: 49.0%–74.0%) in humans. Female animals have higher odds of acquiring *T. gondii* (OR = 1.76; 95% CI: 1.56–2.00) than males. Similarly, adult animals were more likely to be exposed to the disease (OR = 2.39; 95% CI: 2.10–2.74) than young age groups. In humans, rural residence (OR = 1.23; 95% CI: 1.07–1.42) and cat ownership (OR = 2.04; 95% CI: 1.79–2.32) were significantly associated with *T. gondii* seropositivity. These results highlight the necessity of targeted monitoring and preventative measures to reduce *T. gondii* exposure in both animal and human populations in Ethiopia.

## Graphical abstract

This graphical abstract summarizes a systematic review and meta‐analysis of *Toxoplasma gondii* in Ethiopia (2012–2025). The study estimated pooled prevalence in ruminants (42.0%) and humans (61.0%) using random‐effects models. Female and adult animals, rural residence, and cat ownership were identified as significant risk factors. The findings emphasize the need for targeted surveillance and preventive measures to reduce toxoplasmosis exposure in both animals and humans in Ethiopia.

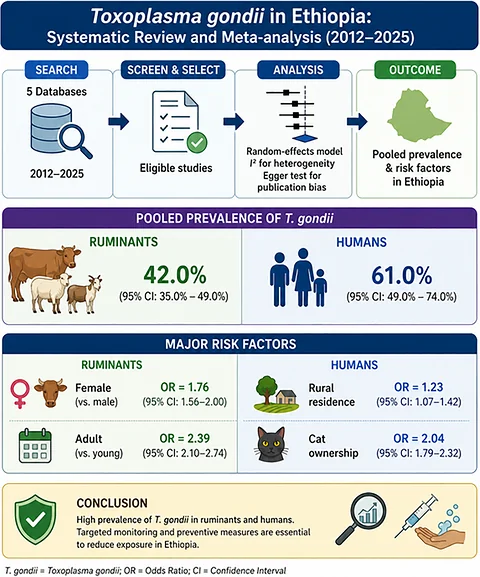

## 1 Introduction

Toxop*lasma gondii* is an apicomplexan obligate intracellular parasite that infects both animals and humans worldwide (Tonouhewa et al. 2017). Felines are the definitive hosts, whereas aquatic and terrestrial animals are the intermediate hosts (Attias et al. 2020; Tagwireyi et al. 2019). The three developmental phases of the parasite's life cycle, namely tachyzoites, bradyzoites, and sporozoites, contribute to different routes of transmission (Attias et al. 2020). These biological characteristics directly shape the diverse pathways through which infection reaches both animal and human populations.

Building on this biological foundation, humans and animals acquire infection by ingestion of food and water contaminated with sporulated oocysts, and by consuming raw or undercooked meat containing tissue cysts. Blood transfusions, organ transplants, and the ingestion of unpasteurized dairy products are also considered less common routes (Dubey et al., 2009; Stelzer et al., 2019).

The clinical consequences of these varied transmission routes are wide‐ranging, affecting both reproductive and systemic health. Infections with *Toxoplasma* have been shown to affect female reproductive systems negatively (Abdoli et al. 2012). In ruminants, the disease may cause miscarriage, stillbirth, foetal death and mummification, early embryonic death and resorption (Edwards and Dubey 2013), resulting in considerable economic losses in the livestock sector (Tagwireyi et al. 2019; Tonouhewa et al. 2017). Toxoplasmosis can be asymptomatic in immunocompetent people, but it can have major negative effects on immunocompromised people, especially those with AIDS and pregnant women (Frimpong et al. 2017; Tagwireyi et al. 2019).

Against this global backdrop of transmission and pathology, local contexts play a critical role in determining infection risk. Environmental, cultural, and management‐related factors impact *T. gondii* infection epidemiology. In Ethiopia, the spread of *T. gondii* is greatly increased due to cultural practices including eating raw or undercooked meat and the close living proximity between humans and cats. Additionally, cats' hunting and scavenging habits contribute to environmental pollution through oocyst shedding, which sustains the cycle of transmission in both humans and animals.

In Ethiopia, published studies have documented considerable variation in seroprevalence of *T. gondii* across host species and regions. According to reports, seroprevalence in ruminants varies from 76% in sheep in the Amhara region (Esubalew et al., 2020) to 8.6% in goats in the Oromia region (Alamerew et al., 2022). Similarly, human seroprevalence has been reported as 18.2% in the Oromia region (Elemo, 2020) and 94.4% in the Southern Nations, Nationalities, and Peoples' Region (Zeleke and Melsew, 2017). This variability likely indicates real differences in host species, geographical setting, diagnostic method and sample size.

Yet despite this growing body of evidence, critical analytical gaps remain. Although earlier reviews have looked at *T. gondii* in specific host groups or subregions, there has not been a thorough synthesis that includes data from ruminants and humans throughout Ethiopia, constraining the conclusions that can be drawn at the national level and explicitly quantifying between‐study variability and related risk variables. A pooled prevalence estimate derived through systematic review and meta‐analysis therefore better identifies gaps in surveillance and priority areas for intervention, while providing a more robust basis for interpreting the cumulative evidence. Therefore, this study aimed to estimate the pooled prevalence of *T. gondii* infection in humans and ruminants in Ethiopia and to identify the major associated risk factors through a systematic review and meta‐analysis.

## 2 Materials and Methods

### 2.1 Study Protocol and Eligibility

This systematic review and meta‐analysis was conducted and reported in accordance with the Preferred Reporting Items for Systematic Reviews and Meta‐Analyses (PRISMA) 2020 guidelines (Page et al. 2021). All prevalence studies of *T. gondii* among ruminants and humans in different regions of Ethiopia using cohort and cross‐sectional observational studies published in English from 2012 to 2025 were included. To prevent duplication of evidence that had previously been reviewed by Gebremedhin and Tadesse (2015), the search period was limited to 2012–2025. Before beginning the review process, the protocol for this systematic review and meta‐analysis was registered in the International Prospective Register of Systematic Reviews (PROSPERO; registration number: CRD420251151270) to guarantee transparency, minimize bias, and prevent unintentional duplication.

### 2.2 Search Strategies and Sources of Information

A comprehensive literature search was performed across five electronic databases: PubMed/MEDLINE, Scopus, Web of Science, ScienceDirect, and Google Scholar to identify studies reporting the prevalence of *T. gondii* in ruminants and humans in Ethiopia. The full search strategy, including all search terms and Boolean operators used for each database, is provided in Supplementary File S1.

### 2.3 Study Selection, Data Extraction, and Quality Assurance

Article screening was conducted using the Condition, Context, and Population (CoCoPo) framework (Munn et al. 2015), whereby titles and abstracts were evaluated against pre‐specified eligibility criteria. (Munn et al. 2015). The condition was the prevalence of *T. gondii*, which is the main outcome of interest; the study population was ruminants and humans, and the context was a study conducted in all regions of Ethiopia. Duplicate articles were removed using Zotero reference manager. The selection process is summarized in the PRISMA 2020 flow diagram (Figure 1).

**FIGURE 1:**
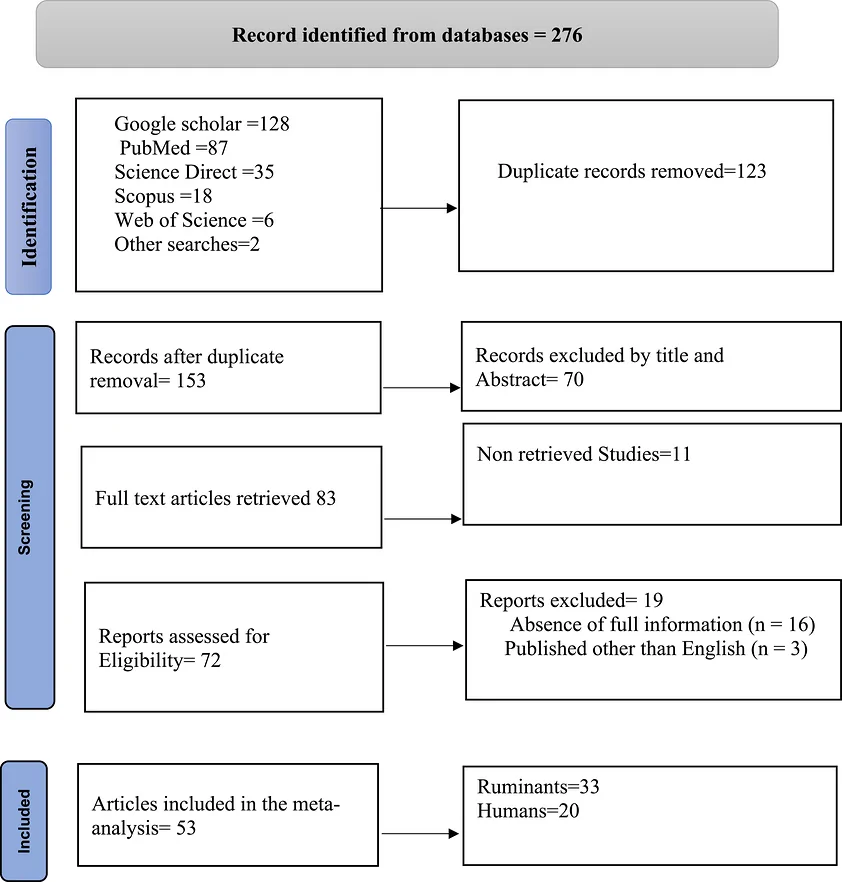
PRISMA flow diagram for study selection (identification, screening, eligibility assessment, and inclusion of studies) in the systematic review and meta‐analysis.

Two independent reviewers screened studies by title and abstract and subsequently assessed the full text of the studies as per the eligibility criteria. Discrepancies between reviewers were resolved through discussion and consensus. From 276 retrieved studies, 53 of them (33 in ruminants and 20 in people) fulfilled the requirements for eligibility and were incorporated into the analysis (Figure 1). Data including author, year of publication, species, region, sample population, sex, age, number of cases, resident, cat ownership, diagnostic tool or method and prevalence were extracted and recorded using Microsoft Excel 2016.

The updated Joanna Briggs Institute (JBI) nine‐item critical appraisal checklist designed for prevalence studies was used to assess methodological quality and risk of bias in each included study, with each item scored as yes (1), no (0), or unclear (0), yielding a maximum possible score of 9 (Barker et al., 2023). Based on the total score out of 9, each study was classified as low quality (score ≤ 3 out of 9), moderate quality (score 4–6 out of 9), or high quality (score ≥7 out of 9), following the classification criteria of Jachmann et al. (2025). All 53 included studies achieved moderate or high‐quality scores (range: 4–9 out of 9); no study was excluded solely on the basis of methodological quality.

### 2.4 Data Analysis

Data were analyzed using R programming language version 4.5.1 (R Core Team 2025). A random‐effects model with a 95% confidence interval (95% CI) was used to compute pooled prevalence estimates. The random‐effects model was selected a priori because the included studies were expected to differ substantially in host species, geographic region, study population characteristics, and diagnostic methods; conditions under which study‐specific true effects are unlikely to be identical and a random‐effects approach is therefore statistically more appropriate than a fixed‐effects model. Between‐study heterogeneity was quantified using the I^2^ statistic, which estimates the proportion of total variation attributable to true heterogeneity rather than sampling error. I^2^ values were interpreted as follows: 0%–25% = low, 26%–50% = moderate, 51%–75% = substantial, and >75% = considerable heterogeneity (Thorlund et al., 2012). The statistical significance of between‐study heterogeneity was evaluated separately using Cochran's Q test, with a *p*‐value < 0.05 indicating statistically significant heterogeneity.

Subgroup analyses were performed to investigate potential sources of heterogeneity. Subgroups were pre‐specified by host species (sheep, goat, cattle, and people), study region (Oromia, Amhara, Tigray, Southern Nations, Nationalities and Peoples' Region, and Addis Ababa City Administration), and diagnostic method (enzyme‐linked immunosorbent assay, modified agglutination test, and latex agglutination test).

Meta‐regression was performed to explore potential sources of heterogeneity, and sensitivity analyses were conducted by excluding studies with high risk of bias. To evaluate publication bias, Egger's regression test and trim‐and‐fill analysis, supplemented by visual inspection of funnel plots, were employed, and a *p*‐value < 0.05 shows the presence of publication bias (Lin and Chu 2018). Potential predictor variables, including sex and age for animals and resident and cat ownership for humans, were recorded and analyzed.

### 2.5 Operational Definition

**Young ruminants** are defined as animals in the juvenile stage of development that have not yet reached sexual maturity: specifically, animals aged less than six months for sheep and goats, and less than one year for cattle.

**Adult ruminants** are defined as animals that have reached sexual maturity: specifically, animals aged six months or older for sheep and goats, and one year or older for cattle.

**Urban peoples** are defined as those residing in administrative capitals (regional, zonal, and woreda capitals), where inhabitants are primarily engaged in non‐agricultural activities (trade, services, and industry) and where basic infrastructure, including electricity, piped water, and road networks, is present.

**Rural peoples** are defined as those residing outside the boundaries of an urban centre, where the primary livelihood is based on subsistence agriculture (crop production and livestock rearing) and access to modern infrastructure (e.g., paved roads, central sewage systems) is limited.

## 3 Result

### 3.1 Literature Search Results

Fifty‐three articles were found eligible for the study. The studies were conducted in different regions of Ethiopia, including Addis Ababa, Amhara, Oromia, Southern Nations, Nationalities, and Peoples (SNNP), and Tigray. Oromia and Amhara regions appear to have a wide range of studies in ruminants and humans, respectively. The studies were performed using ELISA (enzyme‐linked immunosorbent assay), LAT (latex agglutination test), DAT (direct agglutination test), SAT (Sabin–Feldman test), RAT (rapid agglutination test), ICT (immunochromatographic test), and PCR (polymerase chain reaction) to detect the prevalence of *T. gondii* infection in ruminants (Table 1) and humans (Table 2). The total sample size of the included studies was 13,936 (7685 from ruminants and 6525 from humans). Of these, 2802 (38.04%) of ruminants (Table 1) and 3705 (57.22%) of humans (Table 2) were found to be seropositive for *T. gondii* infection.

**TABLE 1:** Prevalence of *T. gondii* in ruminants and description of the incorporated studies in Ethiopia, 2025 (*n* = 33).

Author and study year	Study region	Host species	Diagnostic method	Sample size	Event	Prevalence (%)	95% CI	QAS
Alamerew et al. (2022)	Oromia	Goats	ELISA	35	3	8.60	0.02–0.23	5
Bulla et al. (2023)	Oromia	Sheep	ELISA	156	101	62.20	0.57–0.72	6
Bulla et al. (2023)	Oromia	Goats	ELISA	111	45	40.50	0.31–0.50	8
Esubalew et al. (2020)	Amhara	Sheep	LAT	295	224	76.00	0.71–0.81	4
Esubalew et al. (2020)	Amhara	Goats	LAT	281	182	65.00	0.59–0.70	6
Eticha et al. (2017)	SNNP	Sheep	DAT	80	39	48.75	0.37–0.60	7
Eticha et al. (2017)	SNNP	Goats	DAT	8	6	75.00	0.35–0.97	5
Gebremedhin and Gizaw (2014)	SNNP	Sheep	DAT	124	39	15.00	0.23–0.40	6
Gebremedhin and Gizaw (2014)	SNNP	Goats	DAT	60	9	31.45	0.07–0.27	8
Gebremedhin et al. (2014)	Oromia	Sheep	DAT	305	61	20.00	0.16–0.25	7
Gebremedhin et al. (2014)	Oromia	Goats	DAT	323	50	15.48	0.12–0.20	5
Gebremedhin et al. (2013b)	Oromia	Sheep	ELISA	1130	357	31.59	0.29–0.34	9
Tilahun et al. (2016a)	A/Ababa	Sheep	SAT	194	71	55.91	0.30–0.44	7
Tilahun et al. (2016a)	A/Ababa	Goats	SAT	40	15	11.53	0.23–0.54	7
Tilahun et al. (2016a)	A/Ababa	Cattle	SAT	44	11	12.68	0.13–0.40	6
Debano et al. (2018)	Oromia	Cattle	LAT	391	58	14.80	0.11–0.19	5
Jilo et al. (2021)	Oromia	Sheep	LAT	197	114	57.80	0.51–0.65	7
Jilo et al. (2021)	Oromia	Goats	LAT	203	97	47.80	0.41–0.55	9
Maru et al. (2022)	Amhara	Sheep	SAT	135	69	51.10	0.42–0.60	4
Maru et al. (2022)	Amhara	Goats	SAT	40	29	72.50	0.56–0.85	6
Maru et al. (2022)	Amhara	Cattle	SAT	150	89	59.30	0.51–0.67	7
Tegegne et al. (2016)	Oromia	Sheep	SAT	252	148	58.73	0.52–0.65	7
Tegegne et al. (2016)	Oromia	Goats	SAT	116	64	55.18	0.46–0.64	9
Tilahun et al. (2018)	Oromia	Sheep	ELISA	332	112	33.73	0.29–0.39	8
Tilahun et al. (2018)	Oromia	Goats	ELISA	410	113	27.56	0.23–0.32	8
Tilahun et al. (2018)	Oromia	Cattle	ELISA	326	35	10.74	0.08–0.15	5
Wodage et al. (2023)	Amhara	Sheep	LAT	312	101	32.37	0.27–0.38	4
Yirsa et al. (2024)	Amhara	Sheep	PCR	100	62	62.00	0.52–0.72	5
Yirsa et al. (2024)	Amhara	Goats	PCR	110	58	52.70	0.43–0.62	7
Yirsa et al. (2024)	Amhara	Cattle	PCR	110	60	54.50	0.45–0.64	9
Zewde et al. (2022)	Oromia	Sheep	ELISA	279	155	55.50	0.50–0.61	6
Zewde et al. (2022)	Oromia	Goats	ELISA	109	42	38.53	0.29–0.48	8
Zewdu et al. (2013)	Oromia	Goats	ELISA	927	183	19.74	0.17–0.22	5

Abbreviations: CI, confidence interval; QAS, quality assessment score.

**TABLE 2:** Prevalence of *T. gondii* in humans and description of the incorporated studies in Ethiopia, 2025 (*n* = 20).

Author and study year	Study region	Diagnostic method	Sample size	Event	Prevalence (%)	95% CI	QAS
Achaw et al. (2019)	Amhara	RAT	304	81	26.60	0.22–0.32	6
Adugna et al. (2021)	Amhara	LAT	401	284	70.80	0.66–0.75	7
Agmas et al. (2015)	Amhara	LAT	263	180	68.40	0.62–0.74	5
Elemo (2020)	Oromia	ICT	384	70	18.20	0.14–0.22	4
Endris et al. (2014)	Amhara	RAT	385	341	88.60	0.85–0.92	7
Fenta (2019)	SNNP	ELISA	494	404	81.90	0.78–0.85	6
Gebremedhin et al. (2013a)	Oromia	ELISA	425	346	81.00	0.77–0.85	8
Jula et al. (2018)	SNNP	ELISA	401	96	23.90	0.20–0.28	5
Kassie et al. (2024)	Amhara	ELISA	340	132	38.80	0.34–0.44	9
Negero et al. (2017)	SNNP	LAT	210	159	75.70	0.69–0.81	7
Teweldemedhin et al. (2019)	Tgriy	ELISA	360	128	35.60	0.31–0.41	5
Wodage et al. (2023)	Amhara	LAT	208	67	32.20	0.26–0.39	7
Yohanes et al. (2014a)	SNNP	ELISA	170	150	88.20	0.82–0.93	8
Zeleke and Melsew (2017)	SNNP	ELISA	270	255	94.40	0.91–0.97	6
Zemene et al. (2012)	Oromia	ELISA	201	168	83.60	0.78–0.88	8
Abamecha and Awel (2016)	SNNP	ELISA	232	198	85.30	0.80–0.90	5
Awoke et al. (2015)	Amhara	LAT	384	71	18.50	0.15–0.23	7
Mulugeta et al. (2020)	Amhara	LAT	233	158	67.80	0.61–0.74	4
Tilahun et al. (2016b)	Oromia	DAT	354	233	65.80	0.61–0.71	8
Yohanes et al. (2014b)	SNNP	ELISA	232	184	79.30	0.74–0.84	5

Abbreviations: CI, confidence interval; QAS, quality assessment score.

### 3.2 Overall Pooled Prevalence and Heterogeneity

The random‐effects model in this meta‐analysis revealed a 42.0% (95% CI: 35.0–49.0) overall pooled prevalence of *T. gondii* in ruminants (Figure 2) and 61.0% (95% CI: 49.0–74.0) in humans (Figure 3). Considerable heterogeneity was observed among ruminant studies (*I* ^2^ = 97.8%; *Q*‐test = 1426.37, df = 32) and human studies (*I* ^2^ = 99.4%; *Q*‐test = 3144.70, df = 19), indicating substantial between‐study variability. The Cochran's *Q*‐test was statistically significant (*p* < 0.001) for both groups, confirming that heterogeneity exceeded chance variation. Therefore, subgroup analysis, sensitivity analysis and meta‐regression analysis were carried out to identify the sources of heterogeneity.

**FIGURE 2:**
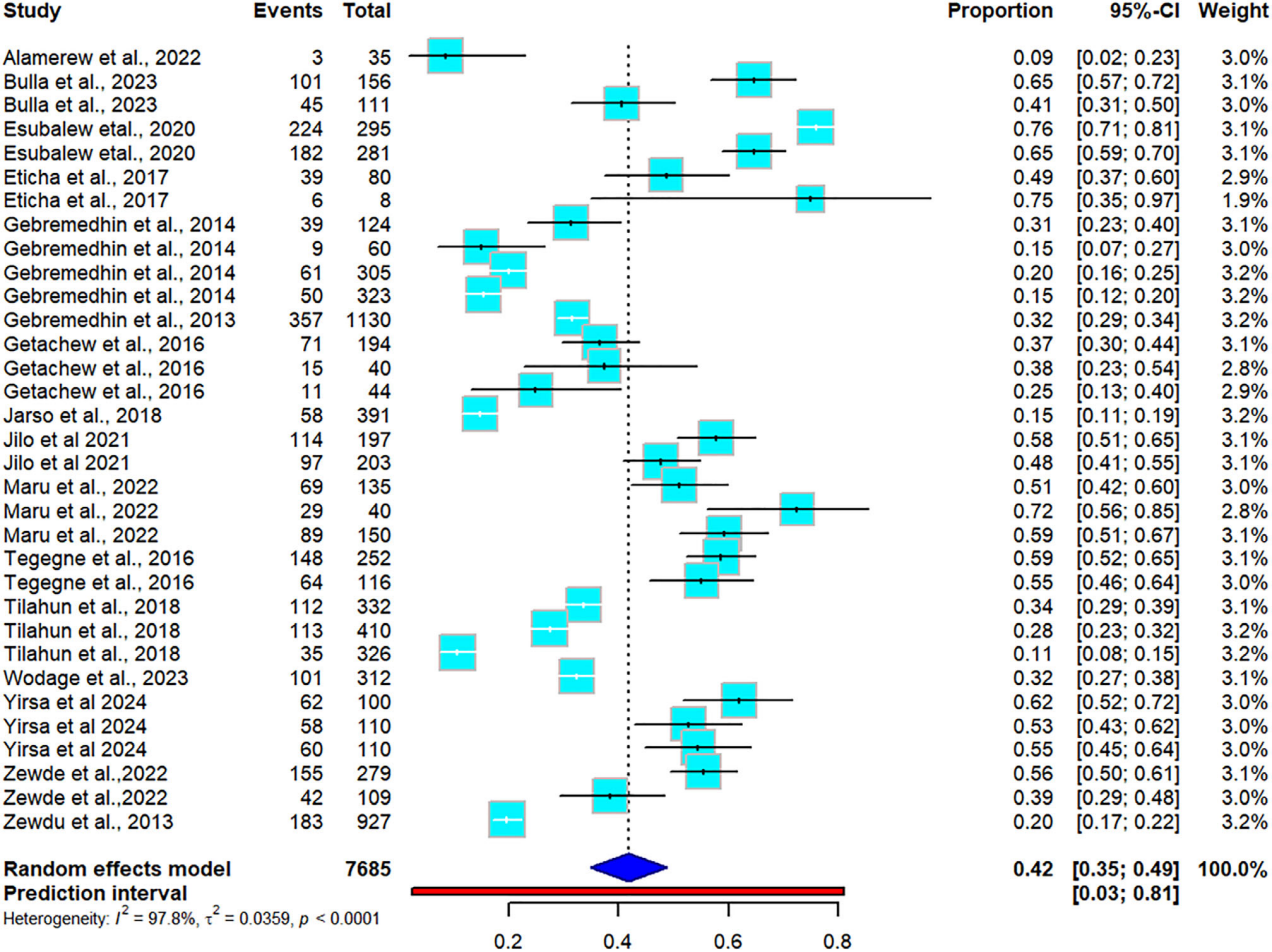
Forest plot for pooled prevalence of *T. gondii* in ruminants in Ethiopia.

**FIGURE 3:**
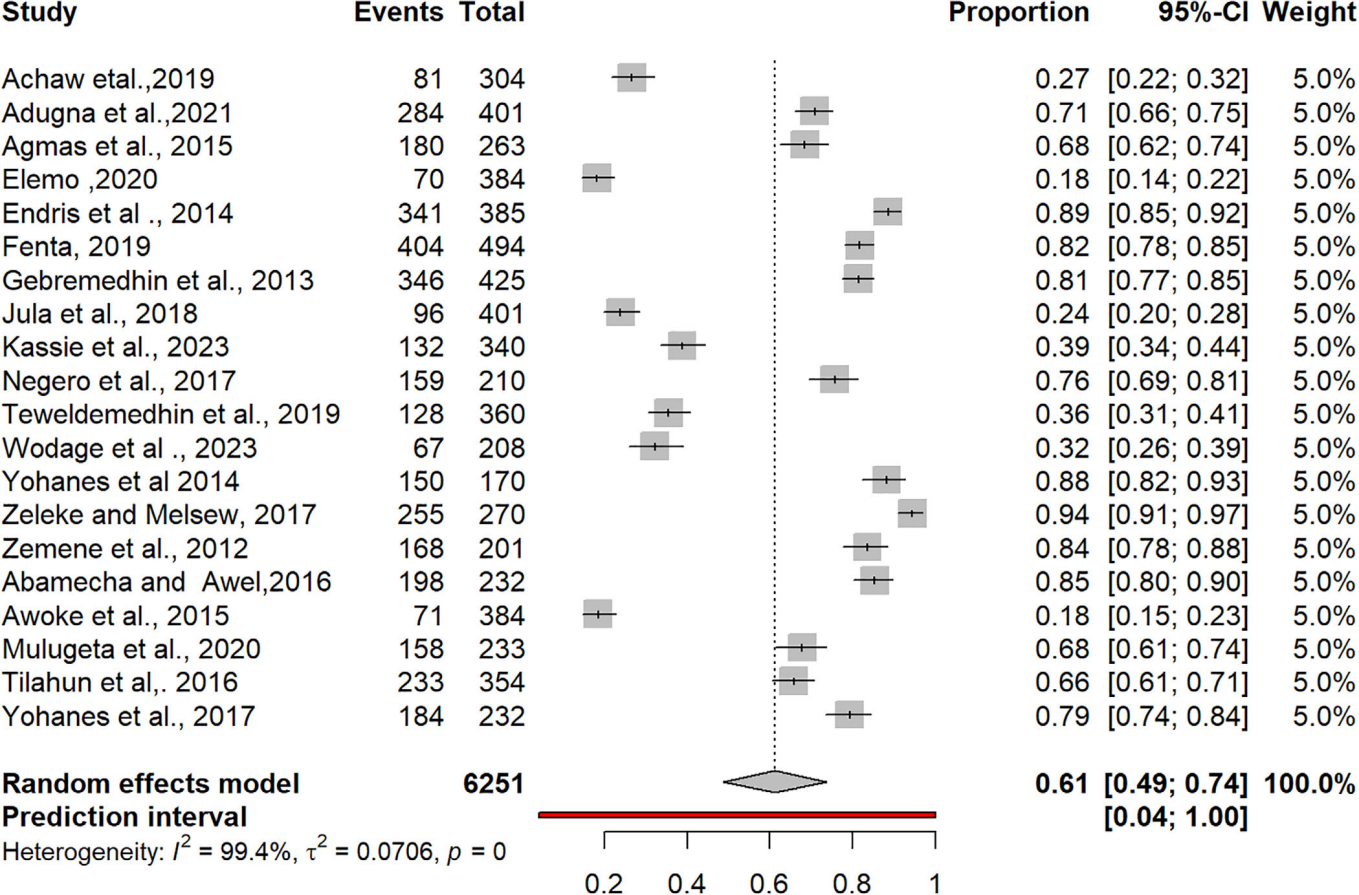
Forest plot for pooled prevalence of *T. gondii* in humans in Ethiopia.

### 3.3 Subgroup Analysis

Subgroup analysis was conducted using the variables including region, species, sample size, publication period, and diagnostic methods. The subgroup analysis by region in ruminants showed the pooled prevalence of toxoplasmosis ranged from 34% in Addis Ababa to 55% in Amhara region, whereas in humans it ranged from 36% in Tigray to 76% in SNNP. The difference between regions was statistically significant (p < 0.0001), highlighting considerable geographic variability in disease burden both in ruminants and humans. Prevalence estimates of toxoplasmosis vary widely depending on the diagnostic method. In farm ruminant studies, higher prevalence is reported in PCR and lower prevalence in DAT, but in humans the ELISA appears to yield the highest prevalence estimate, while LAT shows the lowest prevalence among the diagnostic methods utilized. The prevalence rate between diagnostic methods is statistically significant (*p* < 0.001) in both ruminant and human studies.

A positive temporal trend in pooled prevalence was observed in ruminant studies (52% in studies published ≤2018 vs. 31% in those published >2018), whereas a negative temporal trend was observed in human studies (75% in studies published ≤2017 vs. 44% in those published >2017). The difference between the two time‐based subgroups was statistically significant in both groups (*p* < 0.001). Substantial heterogeneity remained within each subgroup (*I* ^2^ > 94%), suggesting that additional unmeasured factors may have contributed to the observed prevalence variation (Table 3). Different cutoff points for study period and sample size were applied for animal and human studies to ensure an adequate number of studies within each subgroup, thereby improving the statistical power and stability of the pooled estimates.

**TABLE 3:** Subgroup analysis of farm animal and human toxoplasmosis based on different risk factors, Ethiopia.

	Farm animal studies				Human studies			
Variables	Category	Pooled prevalence (95% CI)	*I* ^2^	*p*‐value	Category	Pooled prevalence (95% CI)	I^2^	*p*‐value
Region	Addis Ababa	34.0 (19.0–48.0)	24.2	0.27	Amhara	51(30.0–73.0)	99.3	<0.001
	Amhara	55.0(45.0–64.0)	95.0	<0.001	Oromia	62 (1.4–100.0)	99.5	<0.001
	Oromia	35.0 (26.0–45.0)	97.7	<0.001	SNNP	76 (54.0–97.0)	99.3	<0.001
	SNNP	39.0(01.0–76.0)	90.1	<0.001	Tigray	36 (31.0–41.0)	—	—
Study period	>2018	31.0(22.0–39.0)	95.9	<0.001	>2017	44.0 (26.0–62.0)	99.2	<0.001
	≤2018	52.0(44.0–61.0)	94.9	<0.001	≤2017	75.0 (61.0–89.0)	99.1	<0.001
Sample Size	≤150	45.0(35.0–55.0)	91.8	<0.001	≤300	75.0 (61.0–89.0)	97.9	<0.001
	>150	39.0(29.0–50.0)	97.8	<0.001	>300	50.0 (31–69.0)	99.5	<0.001
Species	Sheep	47.0 (38.0–57.0)	97.4	<0.001				
	Goat	40.0 (27.0–52.0)	96.9	<0.001				
	Cattle	15.0 (08.0–23)	97.8	<0.001				
Diagnostic Methods	ELISA	33.0 (20.0–46.0)	97.5	<0.001	ELISA	69.0 (51–88.0)	99.3	<0.001
	DAT	29.0 (09.0–49.0)	90.0	<0.001				
	LAT	49.0 (25.0–72.0)	99.0	<0.001	LAT	56.0 (30.0–81.0)	99.0	<0.001
	SAT	50.0 (37.0–62.0)	87.7	<0.001				
	PCR	56.0(44.0–68.0)	03.8	0.35				

### 3.4 Sensitivity Analysis

A leave‐one‐out analysis systematically omits one study at a time from a meta‐analysis to assess the influence of each study on the pooled estimate. Hence, in our study, the leave‐one‐out sensitivity analysis shows that the pooled prevalence of toxoplasmosis remains consistent (41–43% in ruminants and 60–64% in humans) regardless of which study is excluded. No individual study substantially affected the overall estimate or the high heterogeneity, indicating the meta‐analysis results are robust (Figures 4 and 5). However, the consistently high heterogeneity suggests considerable variation across studies.

**FIGURE 4:**
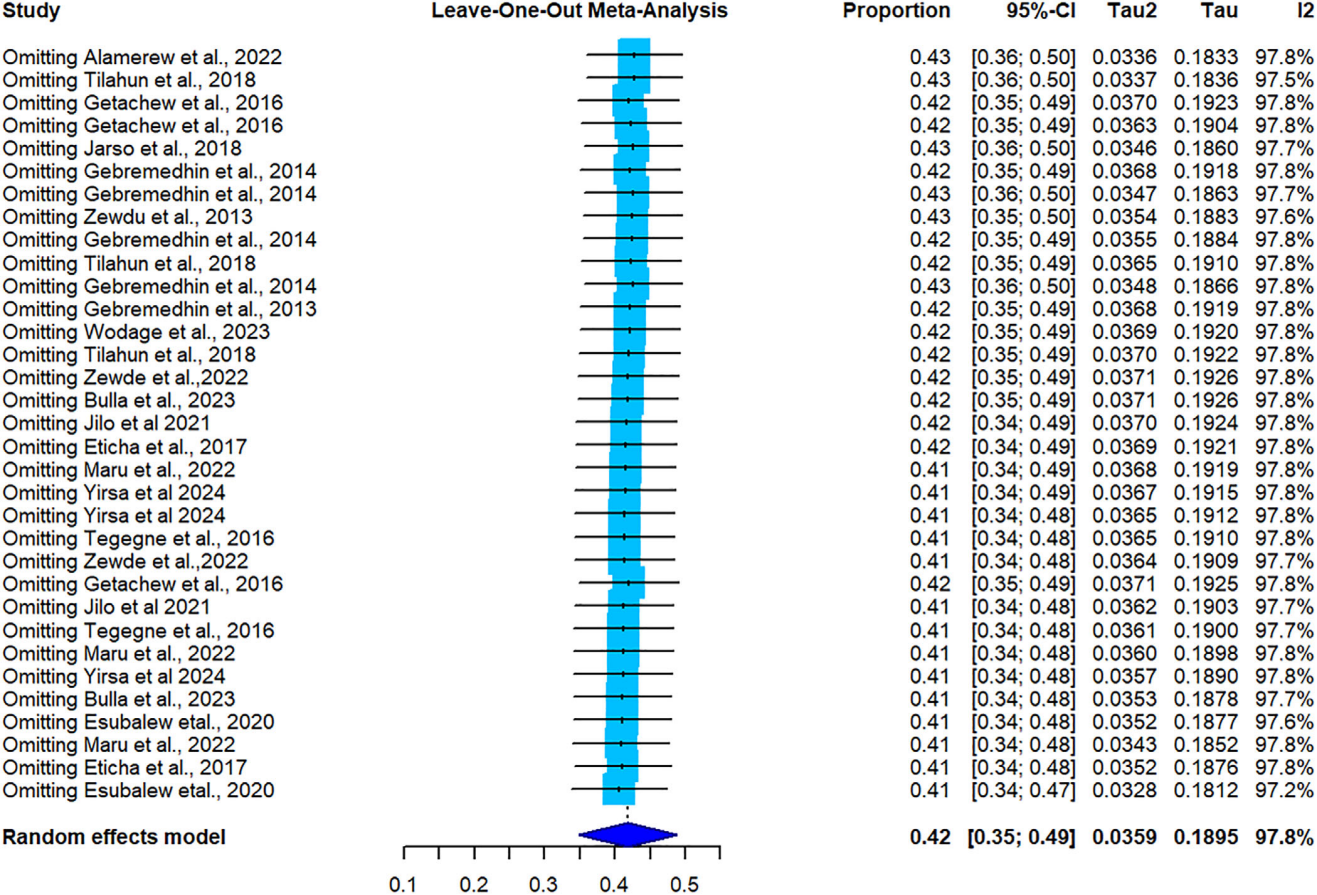
Leave‐one‐out sensitivity analysis in the prevalence of *T. gondii* in ruminants.

**FIGURE 5:**
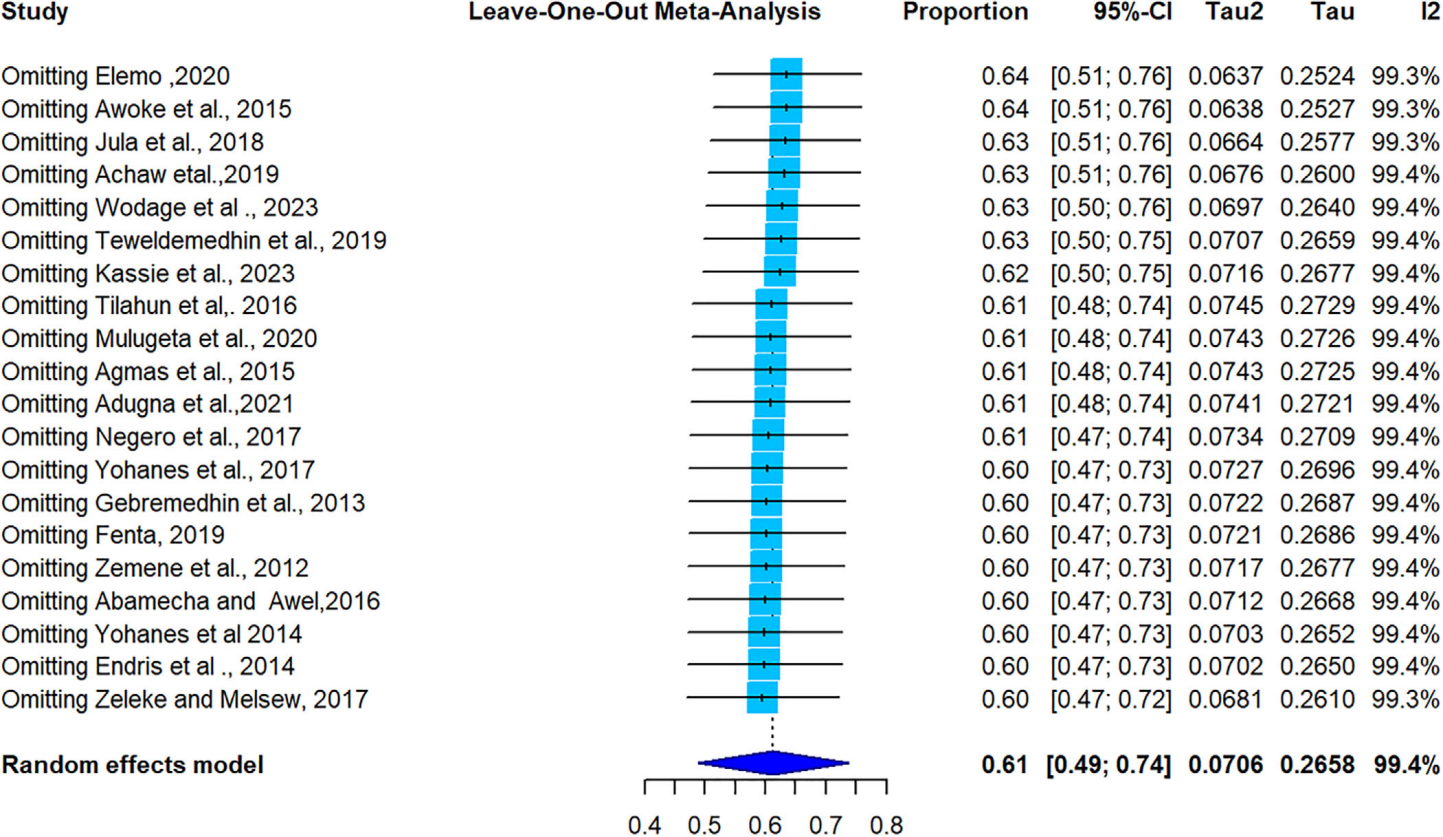
Leave‐one‐out sensitivity analysis in the prevalence of *T. gondii* in humans.

### 3.5 Meta‐Regression Models

#### 3.5.1 Univariable Meta‐Regression

To identify sources of heterogeneity, we performed univariable meta‐regression within a mixed‐effects framework. Sample size and study period were modelled as continuous variables, while study region, animal species, and diagnostic method were treated as categorical variables. Each variable was assessed individually for a linear association with the observed effect sizes.

Of all variables examined, only study period demonstrated a statistically significant association with effect size in both datasets. Study period explained 25.0% of between‐study variance in ruminant studies and 22.15% in human studies (*R* ^2^ = 25.0% and 22.15%, respectively), indicating that roughly one‐quarter of the observed heterogeneity in each dataset is attributable to temporal variation across studies. No other variable reached statistical significance in the univariable analysis (Tables 4 and 5).

**TABLE 4:** Univariable and multivariable meta‐regression analysis of prevalence of *T. gondii* in ruminants in Ethiopia.

		Univariable meta‐regression			Multivariable meta‐regression		
Model	Categories	*R* ^2^ (%)	Estimate (95% CI)	*p*‐value	Estimate (95% CI)	*p*‐value	R^2^ (%)
Study period		25.22	0.0269 (0.0098–0.0440)	0.003	0.0128 (−0.017 to 0.04)	0.378	34.40
Sample size		5.68	−0.0002 (−0.0005 to 0.0000)	0.097	−0.0001 (−0.0005 to 0.00)	0.380	
Species	Cattle	1.89	r		r		
	Sheep		0.1446 (−0.0607 to 0.3498)	0.161	0.1609 (−0.0217 to 0.34)	0.080	
	Goat		0.0696 (−0.1370 to 0.2762)	0.495	0.0818 (−0.1007 to 0.26)	0.370	
Region	Addis Ababa	23.20	r		r		
	Amhara		0.2513 (0.0101−0.4925)	0.041	0.1424 (−0.1622 to 0.44)	0.350	
	Oromia		0.8517 (−0.2058 to 0.2476)	0.852	−0.0080 (−0.2590 to 0.2)	0.950	
	SNNP		0.0556 (−0.2267 to 0.3379)	0.690	0.0057 (−0.2665 to 0.27)	0.970	
Detection method	DAT	16.31	r				
	ELISA		0.0201 (−0.1752 to 0.2154)	0.835			
	LAT		0.1781 (−0.0387 to 0.3948)	0.104			
	SAT		0.1860 (−0.0387 to 0.3948)	0.075			
	PCR		−0.1340 (−0.0200 to 0.3920)	0.404			

Abbreviations: CI, confidence interval; r, reference group.

**TABLE 5:** Univariable and multivariable meta‐regression analysis of prevalence of *Toxoplasma gondii* in humans in Ethiopia.

		Univariable meta‐regression			Multivariable meta‐regression		
Model	Categories	*R* ^2^ (%)	Estimate (95% CI)	*p*‐value	Estimate (95% CI)	*p*‐value	R^2^ (%)
Study period		22.15	−0.0438 (−0.08 to −0.07)	0.021	−0.0388 (−0.6799 to 0.4153)	0.610	
Sample size		02.02	−0.0008 (−0.0022 to 0.0006)	0.254	−0.0001 (−0.0015 to 0.00)	0.900	19.65
Region	Tigray	06.32	r		r		19.65
	Amhara		0.1594 (−0.4213 to 0.7401)	0.569	0.0553 (−0.4852 to 0.5959)	0.830	
	Oromia		0.2668 (−0.3452 to 0.8788)	0.369	0.0650 (−0.5272 to 0.65)	0.820	
	SNNP		0.3999 (−0.1853 to 0.985)	0.167	0.2034 (−0.3620 to 0.768)	0.450	
Diagnostic Method	DAT		r				
	ELISA	00.00	0.0344 (−0.565 to 0.634)	0.900			
	ICT		−0.4759 (−1.284 to 0.332)	0.230			
	LAT		−0.1026 (−0.720 to 0.515)	0.730			
	RAT		−0.0813(−0.781 to 0.618)				

Abbreviations: CI, confidence interval; r, reference group.

#### 3.5.2 Multivariable Meta‐Regression

In the multivariable model, study region, diagnostic method, sample size, and study period were assessed simultaneously. Before fitting the model, collinearity among categorical predictors was examined using the Goodman–Kruskal Gamma coefficient. Study region and diagnostic method were found to be nearly perfectly collinear (γ = −0.922), meaning that these two variables did not vary independently across studies; diagnostic methods were largely determined by the geographic region in which each study was conducted. Including both variables in the same model would make it impossible to disentangle their individual contributions to heterogeneity, resulting in unstable and uninterpretable estimates. Accordingly, the diagnostic method was excluded from the multi‐variable model, and study region was retained as the more substantively meaningful variable.

The final multi‐variable model explained 34.40% of between‐study variance in ruminant studies and 19.65% in human studies (*R* ^2^ = 34.40% and 19.65%, respectively), representing a modest improvement over the univariable model. Although study period was the most consistent predictor across both models, this association should be interpreted cautiously. Study period is a proxy variable that may capture a range of unmeasured temporal changes such as shifts in diagnostic capacity, surveillance intensity, or reporting practices rather than reflecting a true underlying trend in prevalence over time. Furthermore, because a substantial proportion of heterogeneity remains unexplained in both ruminant and human models, study period alone is unlikely to account for all observed variability, and other unmeasured or unreported factors are likely to contribute equally (Tables 4 and 5).

### 3.6 Potential Risk Factors With Positivity for *T. gondii* in Ruminants

#### 3.6.1 Sex

Among the 33 studies incorporated in this meta‐analysis, 16 reported the association between animal sex and *T. gondii* prevalence. Of these, 10 studies identified a statistically significant association between sex and seropositivity, while the remaining six did not yield significant results. Pooled analysis of the 16 studies demonstrated a statistically significant overall association between sex and *T. gondii* seropositivity (OR = 1.76; 95% CI: 1.56–2.00) (Figure 6). These findings indicate that female animals had significantly higher odds of *T. gondii* seropositivity compared with male animals.

**FIGURE 6:**
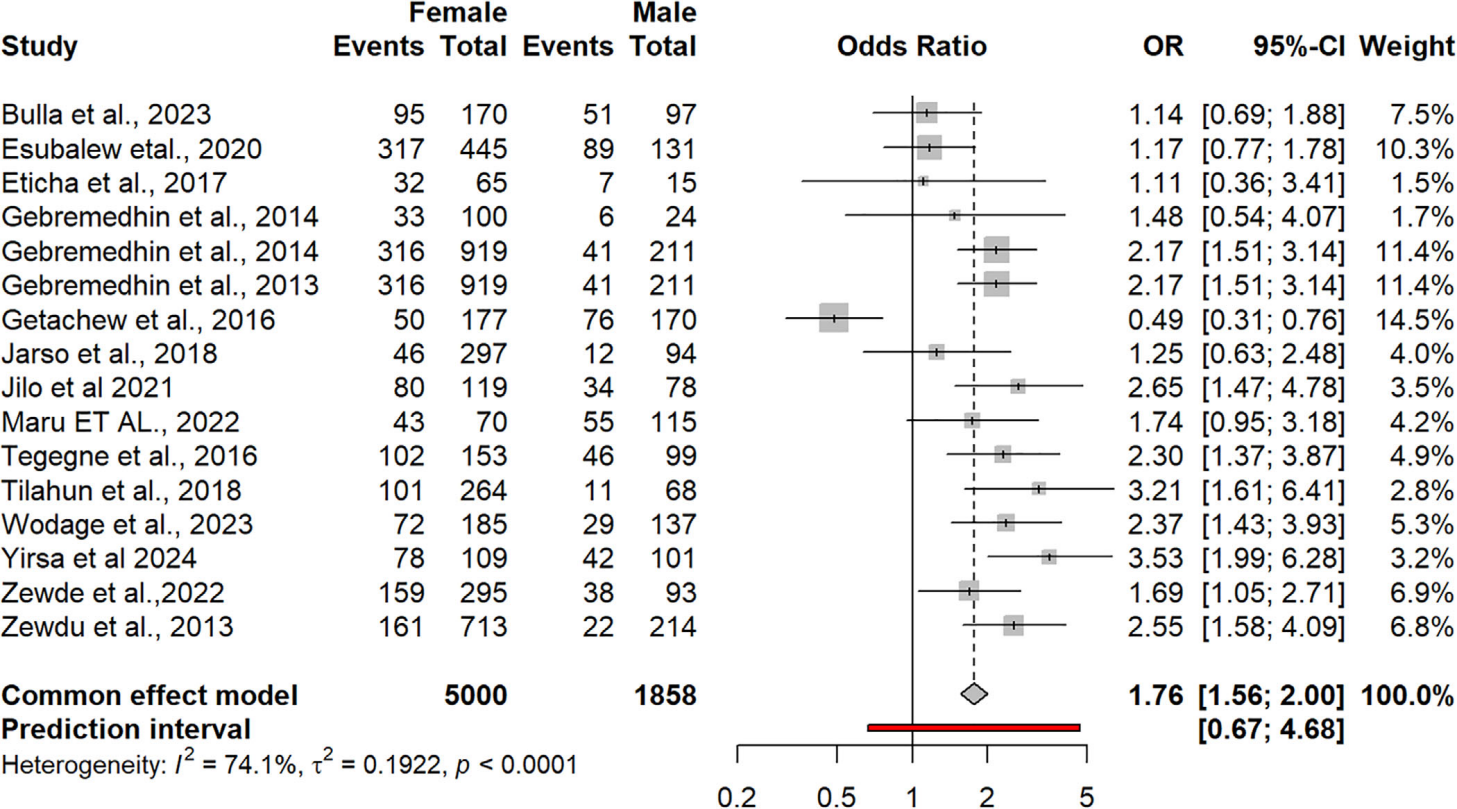
Forest plot for pooled odds ratio of sex and prevalence of *T. gondii* among ruminants in Ethiopia.

#### 3.6.2 Age

Among the 33 studies, 16 examined the relationship between age and *T. gondii* prevalence. Of these 16 studies, 13 identified a statistically significant association between age and seropositivity (Figure 7). Pooled analysis revealed that adult animals had significantly higher odds of *T. gondii* seropositivity compared with young animals (OR = 2.39; 95% CI: 2.10–2.74), indicating that older age was positively associated with *T. gondii* infection.

**FIGURE 7:**
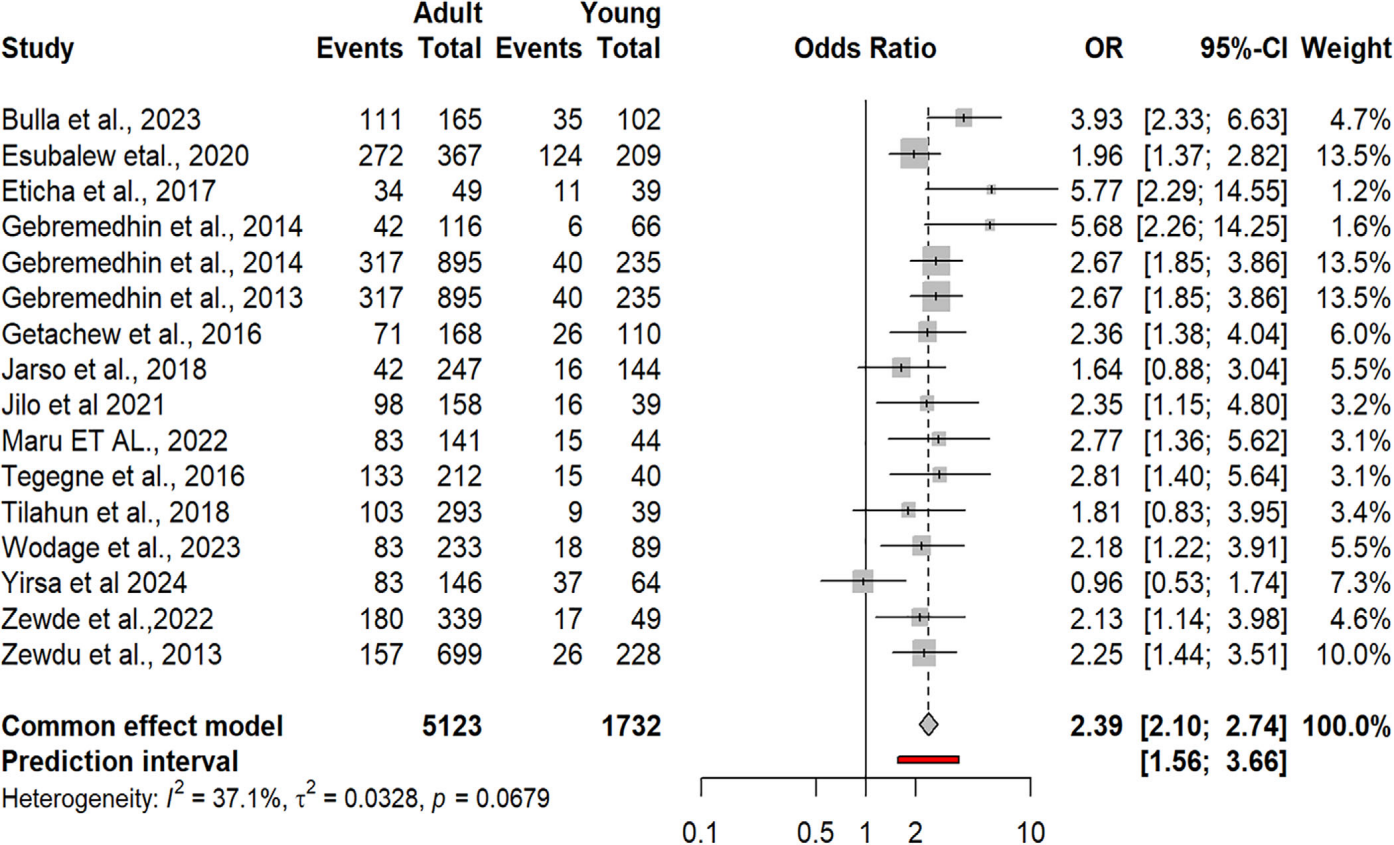
Forest plot for pooled odds ratio of age and prevalence of *T. gondii* among ruminants in Ethiopia.

### 3.7 Potential Risk Factors With Prevalence of *T. gondii* in Humans

#### 3.7.1 Residence

Among the 20 human studies incorporated in this meta‐analysis, 19 reported the association between place of residence and *T. gondii* prevalence. Of these, five studies identified a statistically significant association between residence and seropositivity, while the remaining 14 did not yield significant results. Pooled analysis of the 19 studies demonstrated a statistically significant overall association between rural residence and *T. gondii* prevalence (OR = 1.23; 95% CI: 1.07–1.42) (Figure 8), indicating that individuals residing in rural areas had higher odds of *T. gondii* seropositivity compared with those in urban areas.

**FIGURE 8:**
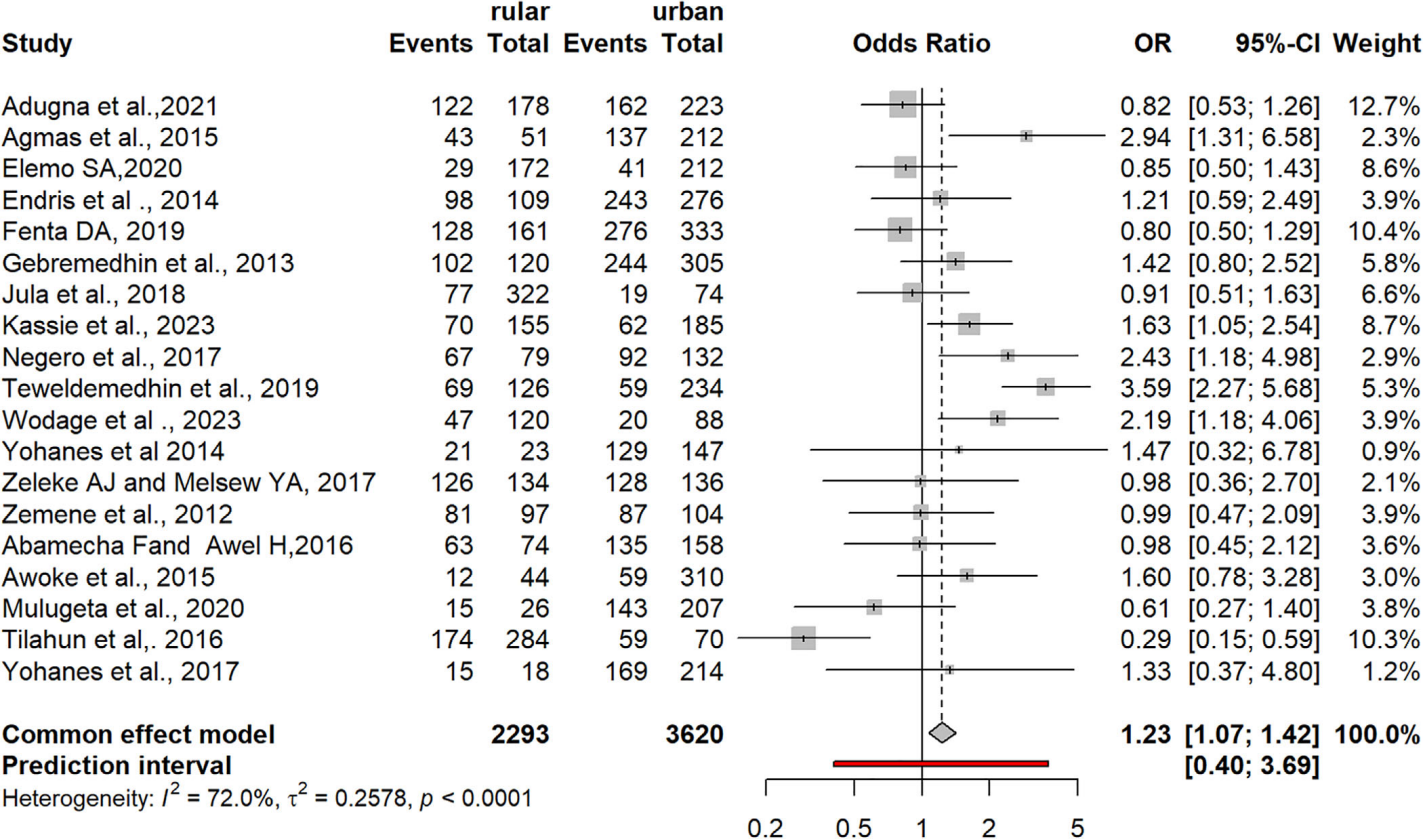
Forest plot shows a meta‐analysis examining the association between residence and toxoplasmosis in humans.

#### 3.7.2 Cat Ownership

Among the 20 human studies, 19 examined the relationship between cat ownership and the prevalence of *T. gondii*. Of these 19 studies, 11 identified a statistically significant relation between cat ownership and the prevalence of *T. gondii* (Figure 9). The overall effect size was: OR = 2.04 (95% CI: 1.79, 2.32). This suggests that cat owners have significantly higher odds of being infected with *T. gondii* compared with non‐cat owners.

**FIGURE 9:**
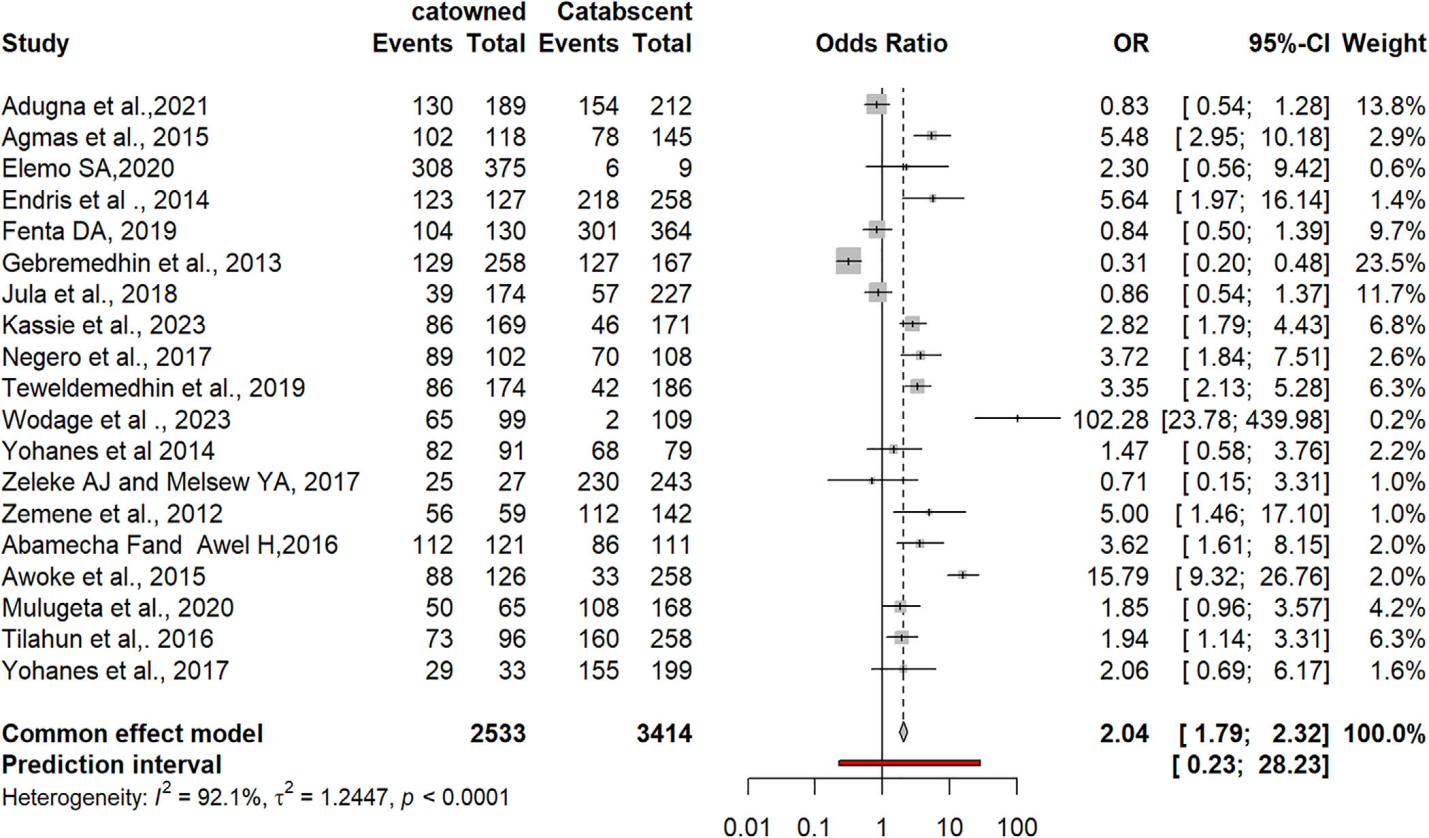
Forest plot shows a meta‐analysis examining the association between cat ownership and toxoplasmosis.

### 3.8 Publication Bias Assessment

Publication bias was assessed using Egger's regression test and trim‐and‐fill analysis, supplemented by visual inspection of funnel plots (Figure 10A,B). Egger's regression test did not provide statistically significant evidence of publication bias in ruminant studies (coefficient = 0.6257; *z* = 1.1131; *p* = 0.2656) or human studies (coefficient = 0.2913; *z* = 1.4742; *p* = 0.1404), suggesting that small‐study effects were not statistically demonstrable. However, because Egger's test has limited power with small numbers of studies and cannot detect all forms of asymmetry, the non‐significant result does not entirely exclude the possibility of publication bias. To further assess potential asymmetry, trim‐and‐fill analysis was conducted as a sensitivity check. This method estimates the number of potentially missing studies and adjusts the pooled estimate accordingly.

**FIGURE 10:**
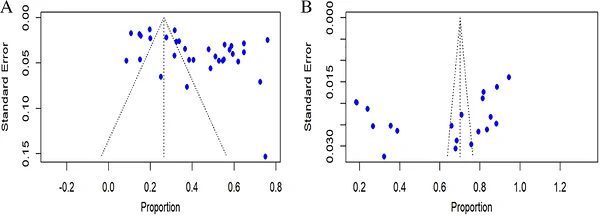
Funnel plot for the pooled prevalence of *T. gondii* in ruminants and humans in Ethiopia.

The trim‐and‐fill analysis imputed 13 hypothetical studies on the left side of the funnel plot for ruminant studies (Figure 11A), indicating possible underrepresentation of studies with lower prevalence estimates. For human studies, two hypothetical studies were imputed on the right side of the funnel plot (Figure 11B), suggesting possible underrepresentation of studies with higher prevalence. It is notable that Egger's test did not reach statistical significance in either group, whereas trim‐and‐fill analysis identified potentially missing studies. This discrepancy is expected: Egger's test evaluates linear funnel plot asymmetry through regression, while trim‐and‐fill is a non‐parametric imputation method that is more sensitive to visual asymmetry. The two approaches are therefore complementary rather than contradictory. After imputation, the adjusted pooled prevalence of *T. gondii* was 26.47% (95% CI: 17.44–35.51) in ruminants and 70.1% (95% CI: 55.38–84.81) in humans, based on 46 studies (33 observed and 13 imputed) and 25 studies (23 observed and 2 imputed), respectively.

**FIGURE 11:**
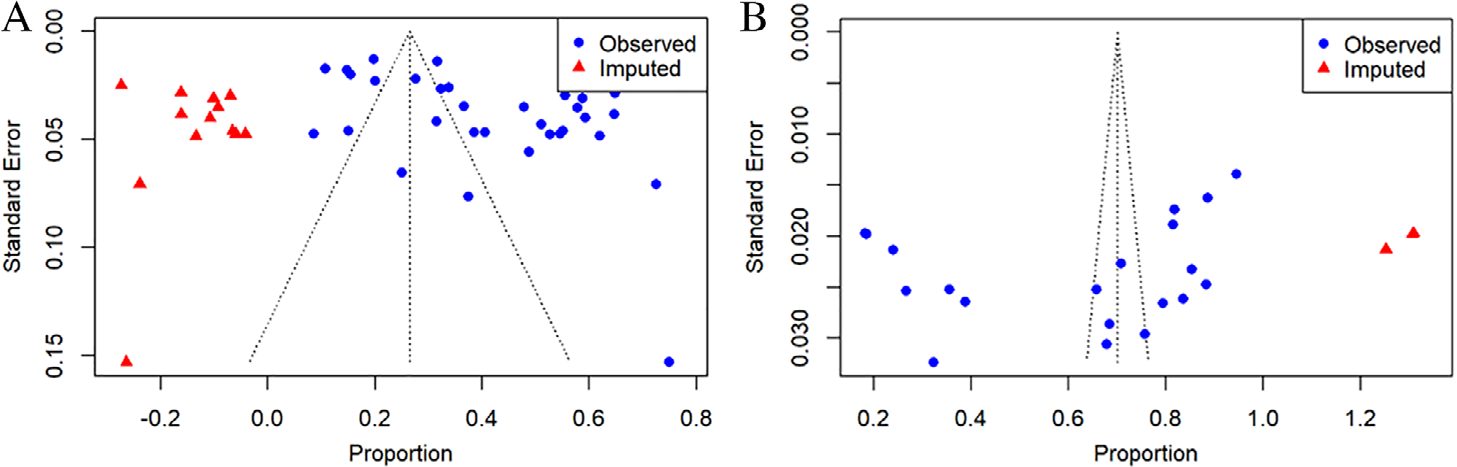
Trim‐and‐fill analysis using imputation of 13 hypothetical studies in ruminants and 2 studies in humans in Ethiopia.

### 3.9 Trends of Animal and Human *T. gondii* Prevalence Over Time

This meta‐regression analysis revealed a significant positive association between study period and the prevalence of toxoplasmosis in ruminants (effect size = 0.0269; 95% CI, 90.0098–0.04400) and a negative association in human populations (effect size = −0.0438; 95% CI, −0.08 to 0.07) (Figure 12A,B)

**FIGURE 12:**
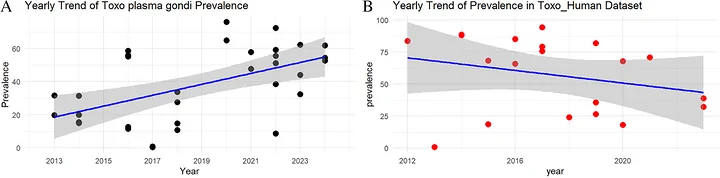
Prevalence of *T. gondii* over time in ruminant and human studies.

## 4 Discussion

In this meta‐analysis, the pooled prevalence of *T. gondii* was 42.0% (95% CI: 35.0–49.0) in ruminants and 61.0% (95% CI: 49.0–74.0) in humans. The ruminant prevalence result is consistent with 43% reported in the Arabian Peninsula (Abdelgadier et al. 2023) and 43.7% in Egypt (Shaapan et al. 2008) but substantially higher than 23.11% in Pakistan (Irshad et al. 2024), 17.0% in Norway (Stormoen et al. 2012) and 13.3% in a Netherlands study (Deng et al. 2016). On the other hand, the human pooled prevalence report is comparable with 64.3% in the Central African Republic (Bouscaren et al. 2018) and 65.62% in Nigeria (Oboro et al. 2016) but markedly exceeds estimates from European countries such as Spain (21.0%) (Bartolomé Alvarez et al. 2008).

This high prevalence of *T. gondii* in Ethiopia, as compared with European countries, may be related to environmental, agricultural, cultural and socioeconomic factors. Hot and humid climatic conditions of Ethiopia may promote oocyst sporulation and environmental persistence (Afonso et al. 2013; Yan et al. 2016). In addition, the common free‐roaming animal husbandry practices in Ethiopia greatly increase the oocyst contamination of pastures and grazing areas, which increases the risk of animal and human exposure to the parasite. Furthermore, the lower socioeconomic development level of Ethiopia may exacerbate the condition because of inadequate sanitation procedures and restricted access to clean water. In the human population, raw or undercooked meat consumption cultural practices may further increase the risk of infection through ingestion of tissue cysts.

Subgroup analysis revealed that there is a significant difference (*p* < 0.001) in the prevalence of *T. gondii* among the study regions and diagnostic methods. The significant variation in prevalence across study regions and diagnostic methods likely reflects a combination of genuine epidemiological differences and methodological heterogeneity. Regional variation may be driven by differences in climate, livestock management, cat exposure, sanitation, and dietary practices, whereas diagnostic variation may result from differences in the sensitivity and specificity of serological assays used across studies (Zhao et al. 2011).

Meta‐regression analysis showed an increasing trend of *T. gondii* prevalence in ruminants and a decreasing trend in humans over time. Similar trends have been reported in other countries (Dong et al., 2018; Jones et al., 2007; Qamar and Alsayeqh, 2023) However, these findings should be interpreted cautiously because the included studies were cross‐sectional and conducted at different time periods and geographical locations. Differences in environmental exposure, livestock management, and diagnostic methods may have contributed to the observed trends.

Female animals had significantly higher odds of *T. gondii* infection than males. This finding is consistent with previous reports from African countries (Arwa Lachkhem and Wahiba Sakly, 2015; Kamani et al., 2010). The higher prevalence in females may be associated with longer retention of breeding females within herds, which increases the likelihood of exposure to infection over time (Tegegne et al., 2016). Physiological stress during pregnancy and lactation may also increase susceptibility to infection (Dubey, 2004).

Adult animals were more likely to be infected than young animals. The result is consistent with reports from European and Asian countries (Bachan et al., 2018; Gazzonis et al., 2015; Sah et al., 2018). This difference is likely due to prolonged environmental exposure in older animals.

Rural residence and cat ownership were identified as statistically significant risk factors for *T. gondii* seropositivity in humans, consistent with reports from Thailand and China (Nissapatorn et al., 2011; Zhou et al., 2011; Zemene et al., 2012). Individuals residing in rural areas had higher odds of seropositivity compared with urban residents, likely reflecting greater occupational and environmental exposure to oocyst‐contaminated soil and water, increased contact with domestic animals including cats, and reduced access to sanitation infrastructure and clean water (Marques et al., 2008; Munoz‐Zanzi et al., 2016). Cat owners demonstrated substantially higher odds of infection, attributable to the potential for accidental ingestion of oocysts shed in cat faeces through contact with litter, contaminated soil, or household surfaces, a transmission route of particular relevance in settings where close human–animal contact is common.

## 5 Conclusion

This meta‐analysis documents a high burden of *T. gondii* infection in both ruminants and humans in Ethiopia, driven by a combination of environmental, husbandry, and behavioural risk factors. Female sex, adult age, rural residence, and cat ownership were each independently associated with higher odds of seropositivity. These findings highlight the need for integrated One Health surveillance and targeted public health interventions, including promotion of safe meat handling practices, improvement of sanitation infrastructure in rural areas, and population‐level awareness of the zoonotic transmission routes of *T. gondii* in the Ethiopian context. Therefore, based on the above conclusion, the following recommendations are forwarded:

1. Strengthen surveillance and screening programs: Targeted screening programs should be implemented, particularly in the Amhara and SNNP regions, where disease prevalence was found to be highest, to enable early detection and timely intervention
2. Implement targeted educational interventions: Public health education campaigns should be directed towards cat owners and rural communities, emphasizing the importance of avoiding feeding cats raw or undercooked meat, keeping cats indoors, and maintaining proper daily litter box hygiene to reduce environmental oocyst contamination
3. Promote safe food handling practices: Awareness programs focusing on proper cooking of meat and safe food handling practices should be strengthened across all regions to minimize the risk of human infection through food‐borne transmission
4. Conduct further research: Additional studies are warranted to better understand regional differences and temporal trends in *T. gondii* infection, enabling the development of evidence‐based and context‐specific prevention strategies

The high burden of *T. gondii* infection in Ethiopia, affecting both animal and human populations, underscores the urgent need for integrated, multisectoral public health strategies to reduce transmission, mitigate associated reproductive and clinical consequences, and safeguard the health of vulnerable populations

### 5.1 Limitations

Lack of studies from certain regions of Ethiopia, which may result in biased national prevalence estimates of *T. gondii*, is the main limitation of the study. Some factors contributing to the prevalence of *T. gondii* were not included in our study due to insufficient data. Additionally, more than 97% of studies relied on serological tests, which can yield false‐negative or false‐positive results, potentially not reflecting the true burden of *T. gondii* on ruminants and humans. Furthermore, the exclusion of socioeconomic level, water source, and meat consumption habits may limit the study because it restricts the ability to identify determinants and explain variations in prevalence of *T. gondii* infection.

## Author Contributions

**Ambachew Motbaynor Wubaye**: conceptualization, investigation, funding acquisition, visualization, project administration, resource, methodology, data curation, formal analysis, Writing – original draft. **Birhanu Feleke Shitu**: methodology, writing – original draft. **Amare Mebrat Delie**: methodology, formal analysis, writing – review and editing. **Aysheshim Belaineh Haimanot**: data curation, writing – review and editing. **Yezbie Wagaw Ayicheh**: data curation, writing – review and editing. **Adugna Mengesha Mulugeta**: formal analysis, writing – review and editing. **Anley Shiferaw Enawgaw**: data curation, supervision, writing – review and editing. **Yihenew Getahun Ambaw**: funding acquisition, visualization, project administration, resource, methodology, formal analysis, writing – review and editing. **Mequanent Dessie Bitewa**: supervision, writing – review and editing. **Smegnew Gichew Wondie**: conceptualization, methodology. **Tadele Derbew Kassie**: data curation, supervision. **Zinie Abita Mengie**: conceptualization, writing – original draft. **Simegnew Adugna Kallu**: funding acquisition, visualization, project administration, resources, conceptualization, methodology, formal analysis, writing – original draft. **Berhanu Ayana Tafere**: data curation, writing – review and editing. **Tesfaye Shumet Mekonnen**: methodology, formal analysis. **Mikias Getahun Molla**: conceptualization, writing – original draft. **Daniel Mulat Eshetu**: data curation, supervision. **Thomas Kidanemariam Yewodiaw**: methodology, formal analysis, writing – review and editing.

## Funding

The authors have nothing to report.

## Ethic Statement

Ethical approval of the study is not required for this systematic review and meta‐analysis.

## Conflicts of Interest

The authors declare no conflicts of interest.

## Supporting information




**Supporting File 1**: vms371293‐sup‐0001‐SuppMat.docx

## Data Availability

The data are available on reasonable request from the corresponding author.
