# Supplementary material for: Toxoplasma gondii in Ethiopia: A Systematic Review and Meta‐Analysis of Infection Prevalence and Associated Risk Factors in Humans and Animals
Source: Vet Med Sci. 2026 Nov 3;12(6):e71293. doi: 10.1002/vms3.71293 (PMC13731163; doi:10.1002/vms3.71293)
Supplement: Supplementary file 1 [file VMS3-12-e71293-s001.docx]

**Supplementary Material**

**Search Engine**

**PubMed and Web of Science**

((“T.gondii” OR “*Toxoplasmosis*”) AND (“prevalence” OR “seroprevalence” OR “serosurvey” OR “epidemiology” OR “infection”) AND (“humans” OR “man” OR “people” OR “patients” OR “livestock” OR “animals” OR “ruminants” OR “cattle” OR “bovine” OR “goats” OR “caprine” OR “sheep” OR “ovine” OR) AND (“Ethiopia” OR Oromia OR “Amhara” OR “Southern Nations Nationalities and peoples” OR “[Afar](https://www.google.com/search?q=Afar&sca_esv=0667ee56d50f5041&sxsrf=ANbL-n7kdR9mLc9Oz4LCQU4c4kikZ56NlA%3A1774664880417&source=hp&ei=sDzHady3F4K75OUP_t_40A4&iflsig=AFdpzrgAAAAAacdKwGl3-3eDG1gGr7GsObpHJb4bl2Y1&ved=2ahUKEwiE3en0xcGTAxVmC7kGHXtWJzIQgK4QegQIAxAB&uact=5&oq=Name+of+the+Ethiopian+regions&gs_lp=Egdnd3Mtd2l6Ih1OYW1lIG9mIHRoZSBFdGhpb3BpYW4gcmVnaW9uczIGEAAYFhgeMgUQABjvBTIFEAAY7wUyBRAAGO8FMgUQABjvBUiuW1CQB1iAUXABeACQAQCYAfQBoAHuJaoBBjAuMjMuNrgBA8gBAPgBAZgCHqAC3yaoAgrCAgcQIxjqAhgnwgIEECMYJ8ICBRAAGIAEwgIKEAAYgAQYywEYCsICCBAAGIAEGMsBwgIIEAAYFhgeGArCAggQABiABBiiBJgDB_EFpvqIpaRheHaSBwYxLjIzLjagB7SPAbIHBjAuMjMuNrgH2CbCBwYxLjIxLjjIB02ACAE&sclient=gws-wiz&mstk=AUtExfBWCsZ1Gnz0iUr5ELS8nyM6cHoGPLC2Q2HtoYiQkBFXHrJ7s3V64-GaH1FBniTe4-Kk0yWxo6TzSc6rJ4KVMGTdgWHlfN1HvbcxvWvvykAN5--JW2sXD6IwMYcuUltl-94kM7oWapItyYA_Eq441U-vasYSr4nTBh_QzBurTe8amlw&csui=3)” OR “[Benishangul-Gumuz](https://www.google.com/search?q=Benishangul-Gumuz&sca_esv=0667ee56d50f5041&sxsrf=ANbL-n7kdR9mLc9Oz4LCQU4c4kikZ56NlA%3A1774664880417&source=hp&ei=sDzHady3F4K75OUP_t_40A4&iflsig=AFdpzrgAAAAAacdKwGl3-3eDG1gGr7GsObpHJb4bl2Y1&ved=2ahUKEwiE3en0xcGTAxVmC7kGHXtWJzIQgK4QegQIAxAF&uact=5&oq=Name+of+the+Ethiopian+regions&gs_lp=Egdnd3Mtd2l6Ih1OYW1lIG9mIHRoZSBFdGhpb3BpYW4gcmVnaW9uczIGEAAYFhgeMgUQABjvBTIFEAAY7wUyBRAAGO8FMgUQABjvBUiuW1CQB1iAUXABeACQAQCYAfQBoAHuJaoBBjAuMjMuNrgBA8gBAPgBAZgCHqAC3yaoAgrCAgcQIxjqAhgnwgIEECMYJ8ICBRAAGIAEwgIKEAAYgAQYywEYCsICCBAAGIAEGMsBwgIIEAAYFhgeGArCAggQABiABBiiBJgDB_EFpvqIpaRheHaSBwYxLjIzLjagB7SPAbIHBjAuMjMuNrgH2CbCBwYxLjIxLjjIB02ACAE&sclient=gws-wiz&mstk=AUtExfBWCsZ1Gnz0iUr5ELS8nyM6cHoGPLC2Q2HtoYiQkBFXHrJ7s3V64-GaH1FBniTe4-Kk0yWxo6TzSc6rJ4KVMGTdgWHlfN1HvbcxvWvvykAN5--JW2sXD6IwMYcuUltl-94kM7oWapItyYA_Eq441U-vasYSr4nTBh_QzBurTe8amlw&csui=3)” OR “[Gambela](https://www.google.com/search?q=Gambela&sca_esv=0667ee56d50f5041&sxsrf=ANbL-n7kdR9mLc9Oz4LCQU4c4kikZ56NlA%3A1774664880417&source=hp&ei=sDzHady3F4K75OUP_t_40A4&iflsig=AFdpzrgAAAAAacdKwGl3-3eDG1gGr7GsObpHJb4bl2Y1&ved=2ahUKEwiE3en0xcGTAxVmC7kGHXtWJzIQgK4QegQIAxAJ&uact=5&oq=Name+of+the+Ethiopian+regions&gs_lp=Egdnd3Mtd2l6Ih1OYW1lIG9mIHRoZSBFdGhpb3BpYW4gcmVnaW9uczIGEAAYFhgeMgUQABjvBTIFEAAY7wUyBRAAGO8FMgUQABjvBUiuW1CQB1iAUXABeACQAQCYAfQBoAHuJaoBBjAuMjMuNrgBA8gBAPgBAZgCHqAC3yaoAgrCAgcQIxjqAhgnwgIEECMYJ8ICBRAAGIAEwgIKEAAYgAQYywEYCsICCBAAGIAEGMsBwgIIEAAYFhgeGArCAggQABiABBiiBJgDB_EFpvqIpaRheHaSBwYxLjIzLjagB7SPAbIHBjAuMjMuNrgH2CbCBwYxLjIxLjjIB02ACAE&sclient=gws-wiz&mstk=AUtExfBWCsZ1Gnz0iUr5ELS8nyM6cHoGPLC2Q2HtoYiQkBFXHrJ7s3V64-GaH1FBniTe4-Kk0yWxo6TzSc6rJ4KVMGTdgWHlfN1HvbcxvWvvykAN5--JW2sXD6IwMYcuUltl-94kM7oWapItyYA_Eq441U-vasYSr4nTBh_QzBurTe8amlw&csui=3)**” OR “**[Harari](https://www.google.com/search?q=Harari&sca_esv=0667ee56d50f5041&sxsrf=ANbL-n7kdR9mLc9Oz4LCQU4c4kikZ56NlA%3A1774664880417&source=hp&ei=sDzHady3F4K75OUP_t_40A4&iflsig=AFdpzrgAAAAAacdKwGl3-3eDG1gGr7GsObpHJb4bl2Y1&ved=2ahUKEwiE3en0xcGTAxVmC7kGHXtWJzIQgK4QegQIAxAL&uact=5&oq=Name+of+the+Ethiopian+regions&gs_lp=Egdnd3Mtd2l6Ih1OYW1lIG9mIHRoZSBFdGhpb3BpYW4gcmVnaW9uczIGEAAYFhgeMgUQABjvBTIFEAAY7wUyBRAAGO8FMgUQABjvBUiuW1CQB1iAUXABeACQAQCYAfQBoAHuJaoBBjAuMjMuNrgBA8gBAPgBAZgCHqAC3yaoAgrCAgcQIxjqAhgnwgIEECMYJ8ICBRAAGIAEwgIKEAAYgAQYywEYCsICCBAAGIAEGMsBwgIIEAAYFhgeGArCAggQABiABBiiBJgDB_EFpvqIpaRheHaSBwYxLjIzLjagB7SPAbIHBjAuMjMuNrgH2CbCBwYxLjIxLjjIB02ACAE&sclient=gws-wiz&mstk=AUtExfBWCsZ1Gnz0iUr5ELS8nyM6cHoGPLC2Q2HtoYiQkBFXHrJ7s3V64-GaH1FBniTe4-Kk0yWxo6TzSc6rJ4KVMGTdgWHlfN1HvbcxvWvvykAN5--JW2sXD6IwMYcuUltl-94kM7oWapItyYA_Eq441U-vasYSr4nTBh_QzBurTe8amlw&csui=3)” OR “[Sidama](https://www.google.com/search?q=Sidama&sca_esv=0667ee56d50f5041&sxsrf=ANbL-n7kdR9mLc9Oz4LCQU4c4kikZ56NlA%3A1774664880417&source=hp&ei=sDzHady3F4K75OUP_t_40A4&iflsig=AFdpzrgAAAAAacdKwGl3-3eDG1gGr7GsObpHJb4bl2Y1&ved=2ahUKEwiE3en0xcGTAxVmC7kGHXtWJzIQgK4QegQIAxAP&uact=5&oq=Name+of+the+Ethiopian+regions&gs_lp=Egdnd3Mtd2l6Ih1OYW1lIG9mIHRoZSBFdGhpb3BpYW4gcmVnaW9uczIGEAAYFhgeMgUQABjvBTIFEAAY7wUyBRAAGO8FMgUQABjvBUiuW1CQB1iAUXABeACQAQCYAfQBoAHuJaoBBjAuMjMuNrgBA8gBAPgBAZgCHqAC3yaoAgrCAgcQIxjqAhgnwgIEECMYJ8ICBRAAGIAEwgIKEAAYgAQYywEYCsICCBAAGIAEGMsBwgIIEAAYFhgeGArCAggQABiABBiiBJgDB_EFpvqIpaRheHaSBwYxLjIzLjagB7SPAbIHBjAuMjMuNrgH2CbCBwYxLjIxLjjIB02ACAE&sclient=gws-wiz&mstk=AUtExfBWCsZ1Gnz0iUr5ELS8nyM6cHoGPLC2Q2HtoYiQkBFXHrJ7s3V64-GaH1FBniTe4-Kk0yWxo6TzSc6rJ4KVMGTdgWHlfN1HvbcxvWvvykAN5--JW2sXD6IwMYcuUltl-94kM7oWapItyYA_Eq441U-vasYSr4nTBh_QzBurTe8amlw&csui=3)**” OR** “[Somali](https://www.google.com/search?q=Somali&sca_esv=0667ee56d50f5041&sxsrf=ANbL-n7kdR9mLc9Oz4LCQU4c4kikZ56NlA%3A1774664880417&source=hp&ei=sDzHady3F4K75OUP_t_40A4&iflsig=AFdpzrgAAAAAacdKwGl3-3eDG1gGr7GsObpHJb4bl2Y1&ved=2ahUKEwiE3en0xcGTAxVmC7kGHXtWJzIQgK4QegQIAxAV&uact=5&oq=Name+of+the+Ethiopian+regions&gs_lp=Egdnd3Mtd2l6Ih1OYW1lIG9mIHRoZSBFdGhpb3BpYW4gcmVnaW9uczIGEAAYFhgeMgUQABjvBTIFEAAY7wUyBRAAGO8FMgUQABjvBUiuW1CQB1iAUXABeACQAQCYAfQBoAHuJaoBBjAuMjMuNrgBA8gBAPgBAZgCHqAC3yaoAgrCAgcQIxjqAhgnwgIEECMYJ8ICBRAAGIAEwgIKEAAYgAQYywEYCsICCBAAGIAEGMsBwgIIEAAYFhgeGArCAggQABiABBiiBJgDB_EFpvqIpaRheHaSBwYxLjIzLjagB7SPAbIHBjAuMjMuNrgH2CbCBwYxLjIxLjjIB02ACAE&sclient=gws-wiz&mstk=AUtExfBWCsZ1Gnz0iUr5ELS8nyM6cHoGPLC2Q2HtoYiQkBFXHrJ7s3V64-GaH1FBniTe4-Kk0yWxo6TzSc6rJ4KVMGTdgWHlfN1HvbcxvWvvykAN5--JW2sXD6IwMYcuUltl-94kM7oWapItyYA_Eq441U-vasYSr4nTBh_QzBurTe8amlw&csui=3)**” OR “**[Tigray](https://www.google.com/search?q=Tigray&sca_esv=0667ee56d50f5041&sxsrf=ANbL-n7kdR9mLc9Oz4LCQU4c4kikZ56NlA%3A1774664880417&source=hp&ei=sDzHady3F4K75OUP_t_40A4&iflsig=AFdpzrgAAAAAacdKwGl3-3eDG1gGr7GsObpHJb4bl2Y1&ved=2ahUKEwiE3en0xcGTAxVmC7kGHXtWJzIQgK4QegQIAxAX&uact=5&oq=Name+of+the+Ethiopian+regions&gs_lp=Egdnd3Mtd2l6Ih1OYW1lIG9mIHRoZSBFdGhpb3BpYW4gcmVnaW9uczIGEAAYFhgeMgUQABjvBTIFEAAY7wUyBRAAGO8FMgUQABjvBUiuW1CQB1iAUXABeACQAQCYAfQBoAHuJaoBBjAuMjMuNrgBA8gBAPgBAZgCHqAC3yaoAgrCAgcQIxjqAhgnwgIEECMYJ8ICBRAAGIAEwgIKEAAYgAQYywEYCsICCBAAGIAEGMsBwgIIEAAYFhgeGArCAggQABiABBiiBJgDB_EFpvqIpaRheHaSBwYxLjIzLjagB7SPAbIHBjAuMjMuNrgH2CbCBwYxLjIxLjjIB02ACAE&sclient=gws-wiz&mstk=AUtExfBWCsZ1Gnz0iUr5ELS8nyM6cHoGPLC2Q2HtoYiQkBFXHrJ7s3V64-GaH1FBniTe4-Kk0yWxo6TzSc6rJ4KVMGTdgWHlfN1HvbcxvWvvykAN5--JW2sXD6IwMYcuUltl-94kM7oWapItyYA_Eq441U-vasYSr4nTBh_QzBurTe8amlw&csui=3)**” OR “**[Addis Ababa](https://www.google.com/search?q=Addis+Ababa&sca_esv=0667ee56d50f5041&sxsrf=ANbL-n7kdR9mLc9Oz4LCQU4c4kikZ56NlA%3A1774664880417&source=hp&ei=sDzHady3F4K75OUP_t_40A4&iflsig=AFdpzrgAAAAAacdKwGl3-3eDG1gGr7GsObpHJb4bl2Y1&ved=2ahUKEwiE3en0xcGTAxVmC7kGHXtWJzIQgK4QegQIBRAB&uact=5&oq=Name+of+the+Ethiopian+regions&gs_lp=Egdnd3Mtd2l6Ih1OYW1lIG9mIHRoZSBFdGhpb3BpYW4gcmVnaW9uczIGEAAYFhgeMgUQABjvBTIFEAAY7wUyBRAAGO8FMgUQABjvBUiuW1CQB1iAUXABeACQAQCYAfQBoAHuJaoBBjAuMjMuNrgBA8gBAPgBAZgCHqAC3yaoAgrCAgcQIxjqAhgnwgIEECMYJ8ICBRAAGIAEwgIKEAAYgAQYywEYCsICCBAAGIAEGMsBwgIIEAAYFhgeGArCAggQABiABBiiBJgDB_EFpvqIpaRheHaSBwYxLjIzLjagB7SPAbIHBjAuMjMuNrgH2CbCBwYxLjIxLjjIB02ACAE&sclient=gws-wiz&mstk=AUtExfBWCsZ1Gnz0iUr5ELS8nyM6cHoGPLC2Q2HtoYiQkBFXHrJ7s3V64-GaH1FBniTe4-Kk0yWxo6TzSc6rJ4KVMGTdgWHlfN1HvbcxvWvvykAN5--JW2sXD6IwMYcuUltl-94kM7oWapItyYA_Eq441U-vasYSr4nTBh_QzBurTe8amlw&csui=3)” OR “[Dire Dawa](https://www.google.com/search?q=Dire+Dawa&sca_esv=0667ee56d50f5041&sxsrf=ANbL-n7kdR9mLc9Oz4LCQU4c4kikZ56NlA%3A1774664880417&source=hp&ei=sDzHady3F4K75OUP_t_40A4&iflsig=AFdpzrgAAAAAacdKwGl3-3eDG1gGr7GsObpHJb4bl2Y1&ved=2ahUKEwiE3en0xcGTAxVmC7kGHXtWJzIQgK4QegQIBRAD&uact=5&oq=Name+of+the+Ethiopian+regions&gs_lp=Egdnd3Mtd2l6Ih1OYW1lIG9mIHRoZSBFdGhpb3BpYW4gcmVnaW9uczIGEAAYFhgeMgUQABjvBTIFEAAY7wUyBRAAGO8FMgUQABjvBUiuW1CQB1iAUXABeACQAQCYAfQBoAHuJaoBBjAuMjMuNrgBA8gBAPgBAZgCHqAC3yaoAgrCAgcQIxjqAhgnwgIEECMYJ8ICBRAAGIAEwgIKEAAYgAQYywEYCsICCBAAGIAEGMsBwgIIEAAYFhgeGArCAggQABiABBiiBJgDB_EFpvqIpaRheHaSBwYxLjIzLjagB7SPAbIHBjAuMjMuNrgH2CbCBwYxLjIxLjjIB02ACAE&sclient=gws-wiz&mstk=AUtExfBWCsZ1Gnz0iUr5ELS8nyM6cHoGPLC2Q2HtoYiQkBFXHrJ7s3V64-GaH1FBniTe4-Kk0yWxo6TzSc6rJ4KVMGTdgWHlfN1HvbcxvWvvykAN5--JW2sXD6IwMYcuUltl-94kM7oWapItyYA_Eq441U-vasYSr4nTBh_QzBurTe8amlw&csui=3)”))

**Scopus**

TITLE-ABS-KEY ("Toxoplasmosis" OR "*T.gondii*")

AND TITLE-ABS-KEY (prevalence)

AND TITLE-ABS-KEY (humans OR ruminants)

AND TITLE-ABS-KEY (Ethiopia)

**ScienceDirect**

Prevalence AND (T.gondii OR Toxoplasmosis) AND (human OR Ruminant OR animal livestock) AND Ethiopia
